# FSCN1 and epithelial mesenchymal transformation transcription factor expression in human pancreatic intraepithelial neoplasia and ductal adenocarcinoma

**DOI:** 10.1016/j.prp.2023.154836

**Published:** 2023-10-02

**Authors:** Hayley T. Morris, William R. Bamlet, Gina L. Razidlo, Laura M. Machesky

**Affiliations:** a Department of Pathology, University Hospital Crosshouse, Kilmarnock KA2 0BE, United Kingdom; b School of Cancer Sciences, University of Glasgow, Glasgow G12 8QQ, United Kingdom; c Division of Clinical Trials & Biostatistics, Department of Quantitative Health Sciences, Mayo Clinic, Rochester, MN 55905, USA; d Division of Gastroenterology & Hepatology, Department of Biochemistry & Molecular Biology, Mayo Clinic, Rochester, MN 55905, USA; e Cancer Research UK Beatson Institute, Garscube Estate, Glasgow G61 1BD, United Kingdom; f Department of Biochemistry, University of Cambridge, Cambridge CB2 1GA, United Kingdom

**Keywords:** Cancer, EMT, Pancreas, PanIN, PDAC

## Abstract

**Background:**

The actin regulatory protein fascin (FSCN1) and epithelial mesenchymal transition (EMT) transcription factor (TF) SLUG/SNAI2 have been shown to be expressed in PDAC and its precursor lesions (pancreatic intraepithelial neoplasia (PanIN), graded 1–3) in *in vitro* and murine *in vivo* studies. Our aim was to investigate the expression of FSCN1 and EMT-TFs and their association with survival in human PanIN and PDAC.

**Methods:**

Expression was investigated *in silico* using TCGA PanCancer Atlas data (177 PDAC samples with mRNA data) and immunohistochemical staining of a tissue microarray (TMA) (59 PDAC patients).

**Results:**

High *FSCN1* expression was associated with poorer overall survival (p = 0.02) in the TCGA data. EMT-TF expression was not associated with survival, however *FSCN1* expression correlated with that of the EMT-TFs *SLUG*/*SNAI2* (rho = 0.49, p < 0.001) and *TWIST1* (rho = 0.52, p < 0.001). TMA IHC showed low expression of SNAI2 and TWIST1 in normal ductal epithelium, while FSCN1 was not expressed. SNAI2 increased slightly in PanIN1–2, then decreased in higher grade lesions. TWIST1 increased in PanIN2–3 and was retained in PDAC. FSCN1 was increasingly expressed from PanIN2 onwards. SNAI2 and TWIST1 expression positively correlated in all grades of PanIN and PDAC (rho = 0.52, p < 0.001). FSCN1 correlated positively with SNAI2 in PanIN1 (rho = 0.56, p < 0.01).

**Conclusions:**

Increased expression of EMT-TFs in low-grade PanIN followed by FSCN1 in PanIN3 and PDAC suggests EMT-TFs may trigger FSCN1 expression and are potential early diagnostic markers. *FSCN1* expression correlated with overall survival in PDAC and may have value as a prognostic marker.

## 1. Introduction

Pancreatic ductal adenocarcinoma (PDAC) is one of a small number of malignancies with no substantial improvement in survival over several decades [1], with a 5-year survival of 8% in the US [2] and most cases presenting in advanced stages [3].

Pancreatic intraepithelial neoplasia (PanIN) is its precursor lesion, defined as “a microscopic, flat or papillary, non-invasive epithelial neoplasm characterised by varying amounts of mucin and degrees of cytologic and architectural atypia” [4]. In clinical practice a two-tier classification system (Baltimore consensus classification) is utilised, separating lesions into low and high-grade [5] although previously a three-tier system (WHO classification) was used [6]. While the current system has greater concordance with disease progression [7,8] and two-tier classification systems generally show less interobserver variability than three-tier systems [9–11], the three-tier system is widely utilised in research, especially in pre-clinical models [12–15] but also in studies on human tissues [16,17] as it better reflects the stepwise progression from benign epithelium, through increasing degrees of dysplasia, to invasive malignancy.

Epithelial to mesenchymal transition (EMT) is a process by which epithelial cells lose characteristics of the epithelial phenotype, such as cell-cell adhesion and apical-basolateral polarity and acquire mesenchymal features such as motility and invasiveness. There is loss of epithelial markers such as E-cadherin and an increase in expression of mesenchymal markers, for example N-cadherin and vimentin. EMT can occur in response to inflammation and as part of the tissue repair process but is also implicated in the development of malignancy [18]. EMT can be triggered via several signalling pathways, including the TGFβ and Wnt pathways [19]. In turn, these pathways utilise a variety of transcription factors (EMT-TFs) such as SNAI1, SLUG/SNAI2, TCF3, TWIST1/2 and ZEB1/2 [20], to alter expression of tissue and cancer-specific proteins. In pancreatic cancer, one such protein is fascin (FSCN1) [12].

Fascin is an actin bundling protein that is widely expressed during embryonic development, but in normal adult tissues is restricted to basal squamous epithelial cells, dendritic cells, endothelium, and cells of mesenchymal and neural crest origin [21,22]. It is expressed in several cancers and in some, including oropharyngeal, colorectal, pancreatic and breast cancer, expression correlates with survival [12,23,24].

SNAI1 and SLUG/SNAI2 belong to the SNAIL family of zinc-finger transcription factors. During development, this family regulates several functions including mesoderm formation and neural differentiation. In adult tissues they regulate several genes which control epithelial cell morphology and function [25]. Increased expression promotes progression of epithelial neoplasms and is associated with poorer survival in gastric and colorectal cancer [26–29].

TWIST1 and TWIST2 belong to the basic helix-loop-helix (bHLH) family of transcription factors. During embryogenesis they regulate cranial suture fusion and neural tube closure [30]. Expression was found to correlate with clinical outcomes in gastric and colorectal cancer [31–33].

Transcription factor 3 (TCF3), also known as E2A, is another bHLH transcription factor, forming heterodimers with tissue-specific bHLH proteins to regulate cell fate during development [26,34]. High expression is associated with poorer survival in cervical [35] and colorectal cancer [36].

The molecular changes driving EMT and how they correspond to phenotypic changes are not fully understood. Previous work by our group [12] explored fascin in a genetically engineered mouse model of metastatic PDAC expressing *Kras*^G12D^ and *Trp53*^R172H^ under the control of *Pdx1*-Cre (widely termed the “KPC” model). PanINs from this model expressed SLUG/SNAI2 and the epithelial marker E-cadherin simultaneously, implying initiation of EMT in pre-malignant lesions, although the phenotype transition had not completed. EMT-TF expression has also been reported in high-grade PanIN and PDAC in human tissues [37,38], although these studies did not examine lower grade PanIN lesions. While FSCN1 and EMT-TF expression has been described in various cancers, there is little research on how they interact. *In vitro* studies have shown FSCN1 expression can be induced by SNAI1, SLUG/SNAI2 [12] and TGF-β, a known EMT trigger [39]. Induction of FSCN1 expression *in vitro* resulted in a mesenchymal phenotype [40] and upregulated SNAI1 [41] while suppression of FSCN1 resulted in downregulation of TWIST1/2 and ZEB1/2 [42]. To our knowledge, the correlation between FSCN1 and EMT-TFs during progression from PanIN to PDAC has not been explored in human tissue.

Here, we investigated FSCN1 and EMT-TF expression in human PanIN and PDAC. We hypothesised higher FSCN1 expression would be associated with lower survival and would correlate positively with EMT-TFs. Several EMT-TFs were first assessed *in silico* to identify those correlating with FSCN1 in PDAC. The expression of these proteins as ductal epithelium progresses to PanIN and PDAC was then investigated by immunohistochemical (IHC) staining of a human pancreatic tissue microarray (TMA).

## 2. Materials and methods

### 2.1. Analysis of publicly available data from cBioPortal

TCGA PanCancer Atlas data were extracted from cBioPortal [43,44]. From the homepage, Pancreatic Adenocarcinoma (TCGA, PanCancer Atlas) was selected. A query was run by selecting “mRNA expression z-scores relative to diploid samples (log RNA Seq V2 RSEM)” and entering the genes of interest (*FSCN1, SNAI1, SNAI2, TWIST1, TWIST2, ZEB1, ZEB2, TCF3*). This returned mRNA expression data for 177 patients. The plot function was used to plot “Putative copy-number alteration” against “mRNA Expression z-scores” and the resulting plot examined to establish the appropriate cut-off for altered gene expression, which was established as 2 standard deviations above the mean for all genes. A new query was then run for each gene using this z-score threshold to define cases of altered gene expression (for example FSCN1: EXP>2). From here the ‘survival curve’ tab raw data for the survival curves were downloaded as a .csv file (see Supplementary materials).

### 2.2. Tissue microarray

Tissue samples were collected between 1990 and 2014 by the Mayo Clinic SPORE in Pancreatic Cancer, under IRB Protocol 354–06, from patients undergoing pancreatic resection for a confirmed PDAC diagnosis who provided written informed consent. A tissue microarray (TMA) was constructed of pancreatic tissue containing PanIN from 70 patients. More than one core was sampled for many patients, however following tissue loss from cutting through the block and the processing and staining of sections, there were a total of 88 surviving tissue cores. Of these, 77 contained ductal tissue which could be graded and from which the results presented below were produced. These 77 cores contained tissue sampled from 59 patients, whose demographics are summarised in Table 1.

### 2.3. Immunohistochemistry

Sections were obtained from the Mayo Clinic Pancreatic Cancer Spore and stained with haematoxylin and eosin and by immunohistochemistry with the following antibodies: FSCN1 mouse monoclonal (Agilent, M3567, 1:150), SNAI2 mouse monoclonal OTIA6 (Novus Biologicals NBP2–03886, 1:400), TWIST1 mouse monoclonal 10E4E6 (Novus Biologicals NBP2–37364, 1:1000).

Full details of the IHC protocols are provided in the Supplementary methods. The number of cores that contained assessable material for each IHC stain are detailed in Supplementary Table S1. REMARK guidelines were followed in reporting this study [45].

### 2.4. Digital analysis

Slides were scanned using the Leica SCN400F (Leica Microsystems) and imported into Halo image analysis platform v3.1 (Indica Labs) including its TMA add-on module. Ducts were graded by a pathologist (HTM), blinded to clinicopathological data, as either normal, PanIN or PDAC. The degree of dysplasia in ducts showing PanIN was classified according to both the three-tiered 2010 WHO classification (PanIN1, PanIN2 or PanIN3) [6] and the two-tiered Baltimore Consensus classification (low grade PanIN or high grade PanIN) [5]. Low grade PanIN according to the Baltimore Consensus incorporates ducts showing features of PanIN1 or PanIN2 as per the 2010 WHO Classification, while high grade PanIN is equivalent to PanIN3. In this paper, where the term “ductal grade” is used, it refers to the grading of ductal epithelium in this study as normal, PanIN (using either the Baltimore or WHO classification) or PDAC.

Histoscores for each IHC antibody were generated using Halo’s CytoNuclear module (for details see Supplementary Methods and Supplementary Table S2).

### 2.5. Statistical analysis

cBioPortal and TMA histoscore results were exported as .csv files and analysed in Python (Python 3.8.1) using Panda, Matplotlib and Seaborn libraries and in R (Studio Version 1.4.1106) using the ggpubr library. Kaplan-Meier survival analysis, Spearman correlation analyses and production of bar charts were performed in Python. 1-way ANOVA with Tukey’s Post-hoc analysis for multiple comparisons was performed in R. Clinical data were analysed using SAS v9.4 on Linux. The multiple core level and ductal grade histoscores for a given subject were averaged to reduce to a single subject (N = 59) level measure. As optimal histoscore cut-offs for each of the antibodies used has not been previously established, a median dichotomisation was used [46] to explore subject level outcomes. Continuous variables were summarised using mean and standard deviation with Wilcoxon rank sum test to assess statistical significance unless otherwise noted. Categorical variables were summarised using count and percentage with Chi-square test to assess statistical significance. Likelihood Ratio Tests (LRT) were used to assess statistical significance for differences in overall survival observed between groups with critical values determined based upon Chi-square distribution (0.95) with 1 degree of freedom. Missing data were excluded. Unless otherwise stated, p values are unadjusted, with p < 0.05 considered statistically significant.

## 3. Results

### 3.1. FSCN1 expression correlates with overall survival and expression of some EMT-TFs in PDAC

**Fig. 1.**
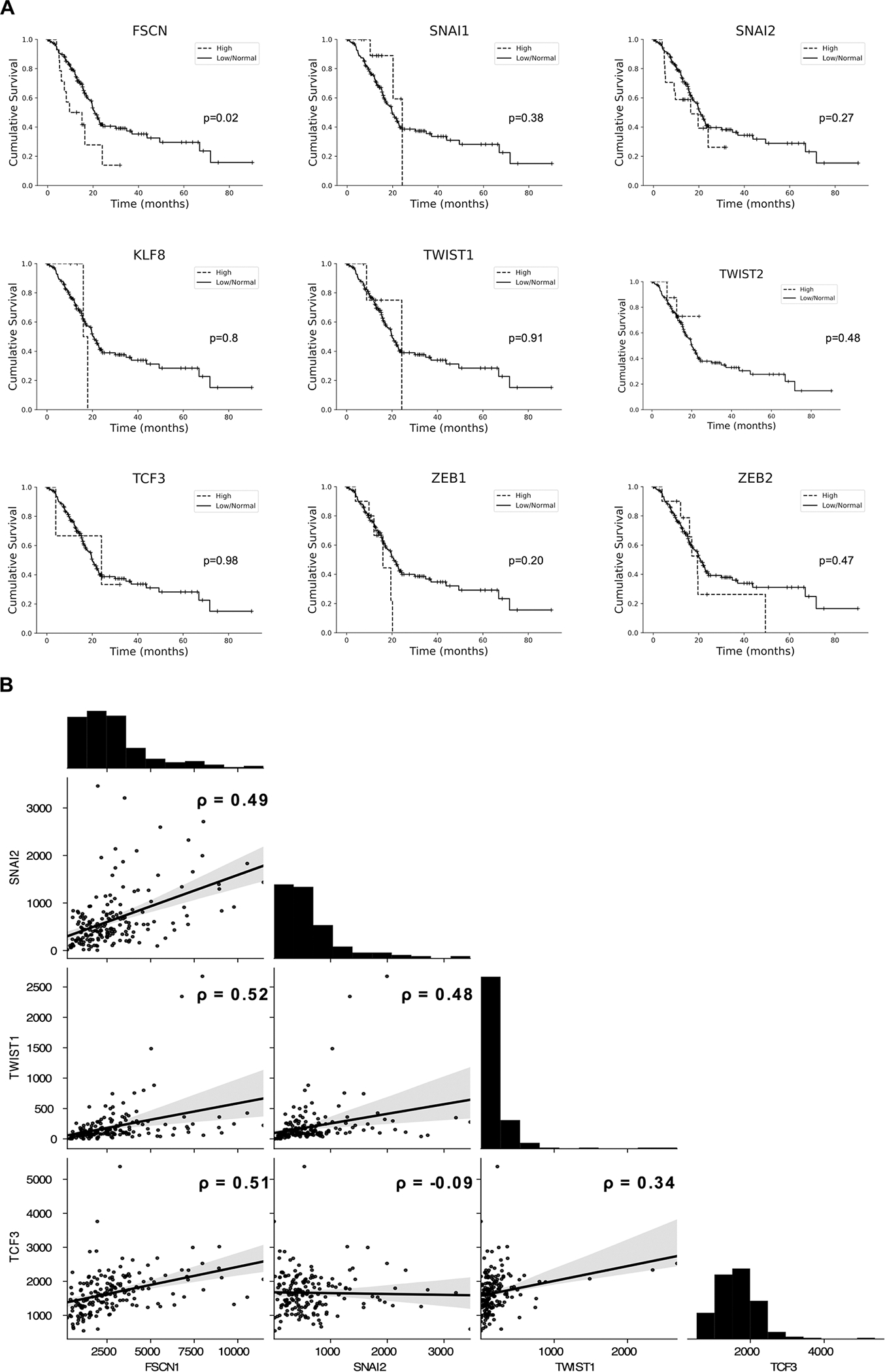
Expression and correlation of FSCN1 and selected EMT transcription factors in human PDAC. (A) Kaplan-Meier curves of survival data extracted from cBioPortal with p-value determined by log-rank test. High expression defined as 2 standard deviations above the mean. n = 177. (B) Histograms of expression and Spearman correlation scatterplots with linear regression lines (grey areas represent the 95% confidence intervals) for correlation between mRNA z-scores (RNA-seq V2 RSEM) of *FSCN1, TWIST1, SLUG/SNAI2* and *TCF3* in human PDAC, with Spearman correlation (rho).

Exploration of mRNA expression data from the TCGA PanCancer Atlas showed high expression of *FSCN1* was associated with lower overall survival of PDAC patients (15.12 vs 20.84 months, p = 0.02) (Fig. 1A). There was no association between survival and expression of any EMT-TFs (Fig. 1A).

Using Spearman’s correlation analysis, there was a moderate and statistically significant correlation between expression of *FSCN1* and *SLUG/SNAI2* (rho = 0.49, p < 0.001), *FSCN1* and *TWIST1* (rho = 0.52, p < 0.001) and *FSCN1* and *TCF3* (rho = 0.51, p < 0.001) (Fig. 1B). None of the other EMT transcription factors showed a significant correlation with *FSCN1* (Fig. S1).

There was a moderate and statistically significant correlation between *TWIST1* and *SLUG/SNAI2* (rho = 0.48, p < 0.001) and a weak and statistically significant correlation between *TWIST1* and *TCF3* (rho = 0.34, p < 0.001). There was no correlation between *SLUG/SNAI2* and *TCF3* (Fig. 1B).

TWIST1 and SLUG/SNAI2 were taken forward alongside FSCN1 to explore protein expression by IHC in the human PanIN TMA. TCF3 immunohistochemistry was also performed but despite successful optimisation on whole slide images, staining on the TMA slides was inadequate for analysis and due to the limited tissue available it was not possible to attempt this with another antibody.

### 3.2. FSCN1 is increased in high-grade PanIN and PDAC

**Fig. 2.**
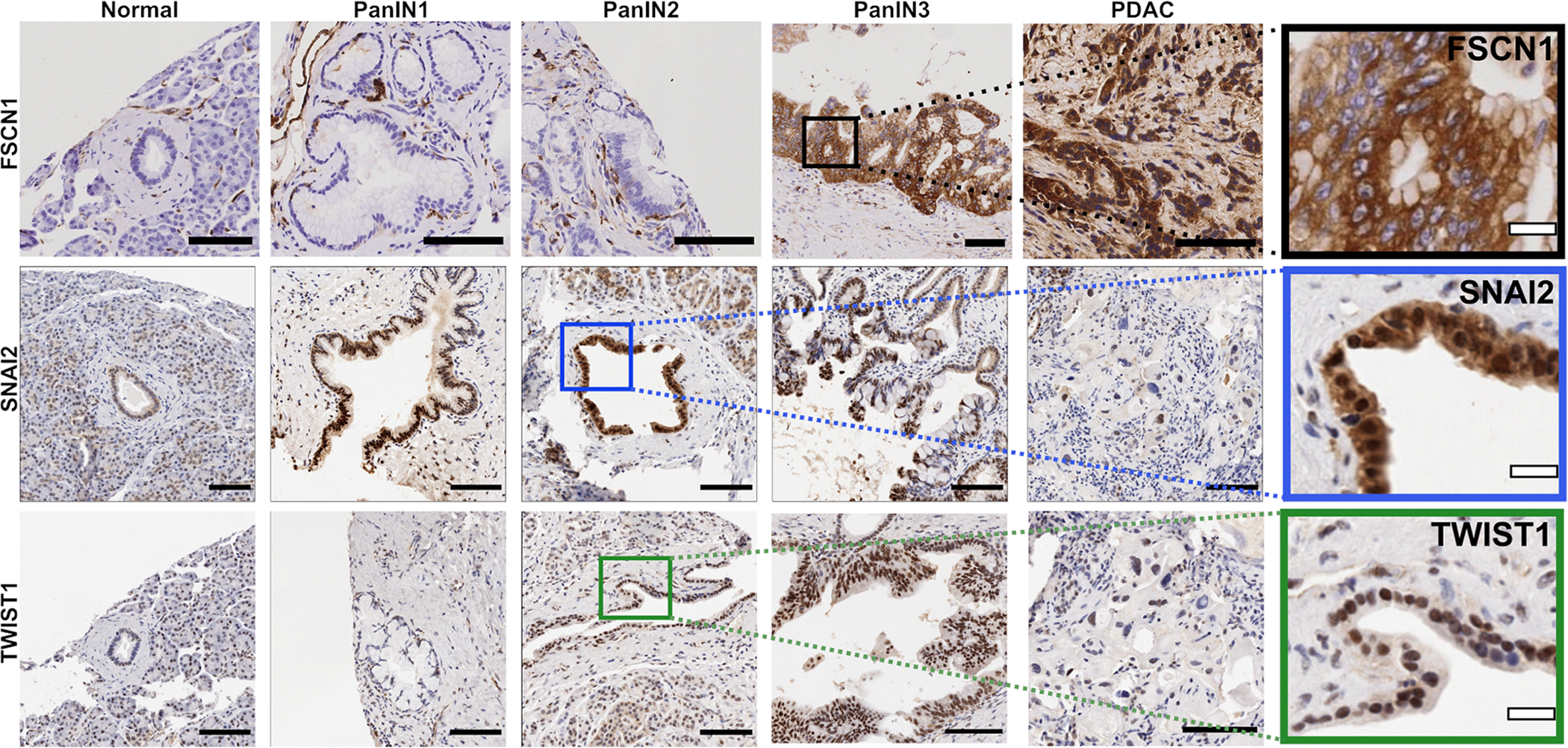
Immunohistochemistry of FSCN1, SLUG/SNAI2 and TWIST1 in human pancreas, PanIN and PDAC. Representative images showing expression of FSCN1 (top row), SLUG/SNAI2 (middle row) and TWIST1 (bottom row) in normal pancreas, ducts graded as PanIN1–3 and PDAC (see Supplementary Table S1 for numbers of cores assessed per grade). PanIN1 and PanIN2 are equivalent to low grade PanIN and PanIN3 equivalent to high grade PanIN in the Baltimore consensus classification. Boxes highlight areas in zoom images. Zoom images (last column) show detail of location of staining for FSCN1, SLUG/SNAI2 and TWIST1 in ductal epithelium. Black scale bars = 100 µm. White scale bars = 20 µm.

FSCN1 expression was localised to the cytoplasm (top row Fig. 2). Consistent with previous studies [12], we found normal ductal epithelium does not express FSCN1. A small number of ducts were classified as showing low (1 +) or moderate (2 +) expression (Fig. S2), which may reflect occasional dendritic cells which lie along the periphery of ducts being counted by the Halo algorithm. Ducts with PanIN1 showed similar staining to normal ducts (Fig. 2, Fig. S2). In PanIN2 there was a small increase in the percentage of 1 + cells (Fig. S2), which correlated with an observed expression of FSCN1 in occasional epithelial cells (Fig. 2). The trend continued in PanIN3 and PDAC with an increase in the number of positive cells and intensity of staining (Fig. 2, Fig. S2).

**Fig. 3.**
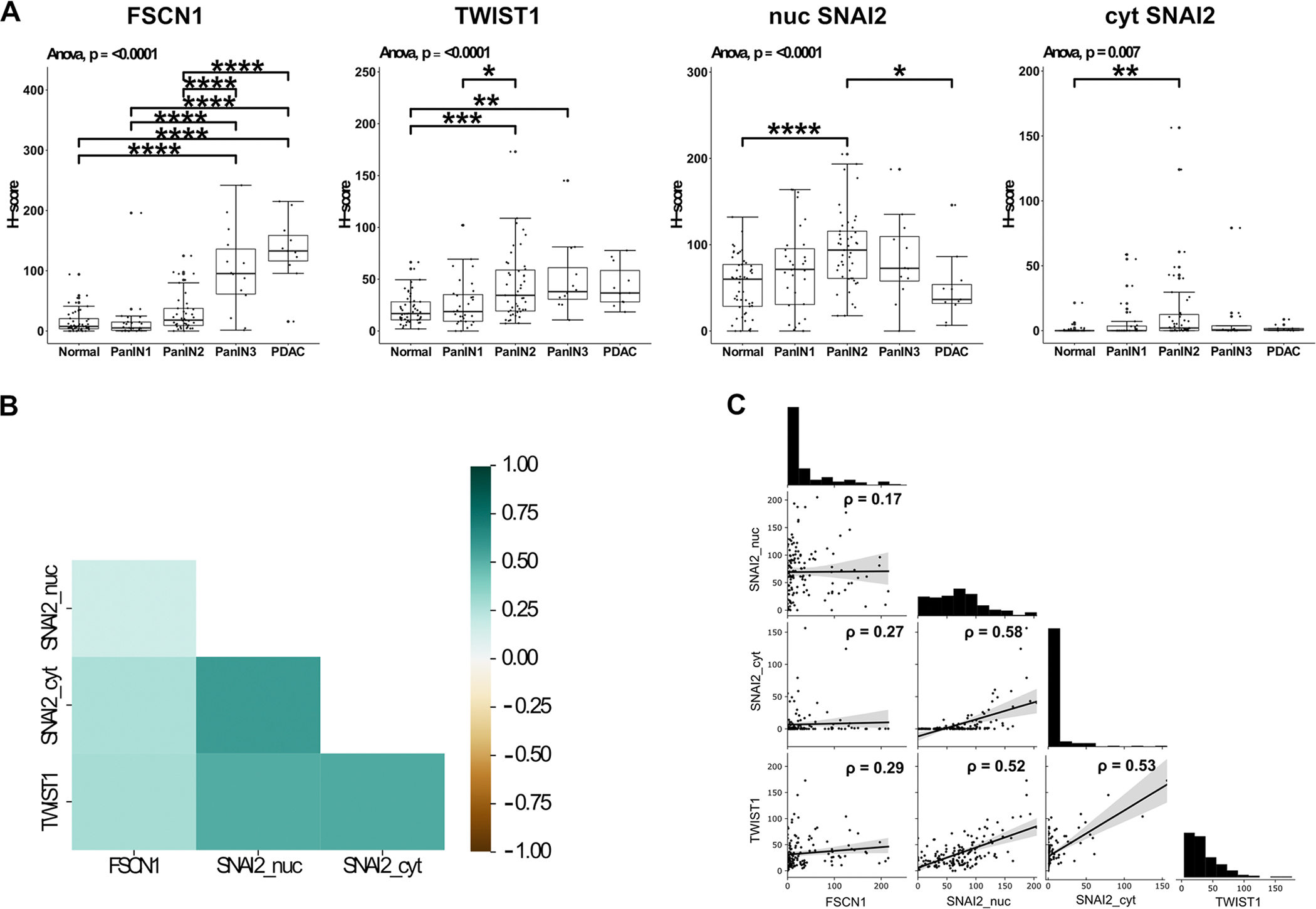
TMA histoscores ANOVA and overall correlation. (A): Tukey boxplots (minimum value, first quartile, median, third quartile, maximum value) of IHC histoscores by duct type with results of ANOVA analysis (n = 11–52, see Supplementary Table S1 for numbers of cores assessed per grade). PanIN1 and PanIN2 are equivalent to low grade PanIN and PanIN3 equivalent to high grade PanIN in the Baltimore consensus classification. Nuc = nuclear, cyt = cytoplasmic. *p < 0.05, * *p < 0.01, * **p < 0.001, * ** *p < 0.0001 (Tukey’s post-hoc test). (B): Heatmap showing correlation of FSCN1, TWIST1 and SLUG/SNAI2 (cytoplasmic and nuclear) histoscores. (C): Histograms of expression and Spearman correlation scatterplots with linear regression lines (grey areas represent the 95% confidence intervals) for correlation between histoscores of FSCN1, TWIST1 and SLUG/SNAI2 with Spearman correlation (rho).

1-way ANOVA showed a statistically significant correlation (p < 0.0001) between FSCN1 histoscore and ductal grade (Fig. 3A). Tukey’s post-hoc analysis showed a significant increase in FSCN1 expression in PanIN3 (mean h-score 100.8) and PDAC (mean h-score 135.1) compared to the other 3 categories (mean h-scores: normal 15.59, PanIN1 16.7, PanIN2 30.17). The differences between normal, PanIN1 and PanIN2 and between PanIN3 and PDAC did not achieve statistical significance (Fig. 3).

Re-grouping the TMA data according to the current diagnostic PanIN classification system into normal, low-grade (equivalent to PanIN1 and 2) and high-grade (equivalent to PanIN3) including PDAC resulted in a statistically significant increase in FSCN1 expression between normal ducts and high-grade lesions and between low grade and high-grade lesions (Fig. S3).

There was no association between FSCN1 expression and any of the patient demographic variables (Supplementary Table S3).

### 3.3. SLUG/SNAI2 expression increases slightly to a peak in PanIN2, then falls in high-grade and invasive ductal epithelial cells

SLUG/SNAI2 staining was predominantly within the nucleus, with weak cytoplasmic expression observed focally (Fig. 2, middle row). We confirmed previous reports that SLUG/SNAI2 is expressed in some ductal epithelial cells [47]; about 50% of cells demonstrated nuclear SLUG/SNAI2, mostly with low intensity (1 +) (Fig. S2). Ducts with PanIN1 showed similar staining to normal ducts, with a small increase in the number of 2 + and 3 + cells (Fig. S2). This trend continued with PanIN2, where 70% of cells expressed SLUG/SNAI2, although mostly still at low intensity. The trend reversed, with lower numbers of positive cells in PanIN3, while PDAC showed a slightly lower level of expression than normal ducts.

1-way ANOVA showed a statistically significant correlation (p < 0.001) between SLUG/SNAI2 nuclear histoscore and grade of ductal epithelium (Fig. 3A). Tukey’s post-hoc analysis showed a significant increase in nuclear SLUG/SNAI2 expression in PanIN2 (mean h-score 94.4) compared to both normal ductal epithelium (53.7) and PDAC (49.0). None of the other inter-group analyses returned a significant correlation (PanIN1 mean h-score 71.0, PanIN3 mean h-score 82.7).

Using the diagnostic classification system as before, there was a statistically significant increase in SLUG/SNAI2 histoscore between normal and low-grade PanIN but no difference between high-grade lesions and either of the other groups (Fig. S3).

In normal ducts, cytoplasmic expression of SLUG/SNAI2 was seen focally (0.5–13% of normal ductal epithelial cells) in 19% of the cores (Fig. S2). The expression in neoplastic ducts mirrored that of nuclear staining (Fig. S2). There was a slight increase in expression in PanIN1, although the staining was predominantly weak (1 +) with some cells showing moderate (2 +) expression. This trend continued with PanIN2, with about 10% of cells showing cytoplasmic staining. As with nuclear staining, the trend reversed in PanIN3 and in PDAC expression was similar to that of normal ductal epithelial cells.

1-way ANOVA showed a statistically significant correlation (p = 0.007) between cytoplasmic SLUG/SNAI2 histoscore and grade of ductal epithelium (Fig. 3A). Tukey’s post-hoc analysis showed a significant increase in cytoplasmic SLUG/SNAI2 expression in PanIN2 (mean h-score 15.5) compared to normal (0.8) ductal epithelium. None of the other inter-group analyses returned a significant correlation (PanIN1 mean h-score 6.9, PanIN3 8.9, PDAC 1.8). Using the new classification system, a similar result was obtained, with increased expression in low-grade PanIN compared to normal ductal epithelium (Fig. S3).

There was no association between level of SNAI2 expression and any of the patient demographic variables (Supplementary Table S3).

### 3.4. TWIST1 expression is increased in PanIN2 and PanIN3 compared to normal ducts and PanIN1

TWIST1 staining was localised to the nucleus (bottom row Fig. 2). Consistent with previous reports [47], we found TWIST1 expression in some normal ductal epithelial cells. This was seen in about 20% of epithelial cells and was predominantly weak (1 +), with a small number of moderate (2 +) cells and occasional cells with strong expression (3 +) (Fig. S2). PanIN1 ducts showed a similar pattern of staining. There was an increase in expression in PanIN2 and PanIN3, with an increase in the number of 1 + and 2 + cells. Similar to SLUG/SNAI2, expression of TWIST1 in PDAC was lower than that of PanIN3.

1-way ANOVA showed a statistically significant correlation (p < 0.0001) between TWIST1 histoscore and grade of ductal epithelium (Fig. 3A). Tukey’s post-hoc analysis showed a significant increase in TWIST1 expression in PanIN2 (mean h-score 44.0) compared to both normal ductal epithelium (21.2) and PanIN1 (26.5). There was a statistically significant difference between PanIN3 (50.7) and normal ductal epithelium. None of the other inter-group analyses returned a significant result (PDAC mean h-score 43.6).

Re-grouping the TMA data as before (Fig. S3), showed a statistically significant increase in h-score between normal ducts and both low-grade and high-grade lesions. There was no difference between h-scores of low-grade and high-grade lesions.

There was no association between level of TWIST1 expression and any of the patient demographic variables (Supplementary Table S3).

### 3.5. FSCN1 and SLUG/SNAI2 expression correlates in PanIN1

The FSCN1 and SLUG/SNAI2 h-scores were compared on a core-by-core basis, with each data “pair” being the average FSCN1 and SLUG/SNAI2 h-scores of all cells of each grade per core.

Considering all epithelial types together, there was no correlation between FSCN1 and nuclear SLUG/SNAI2 h-scores (rho = 0.17, p = 0.053) (Figs. 3B and 3C). There was a weak correlation between FSCN1 and cytoplasmic SLUG/SNAI2 h-scores (rho = 0.27, p < 0.01).

**Fig. 4.**
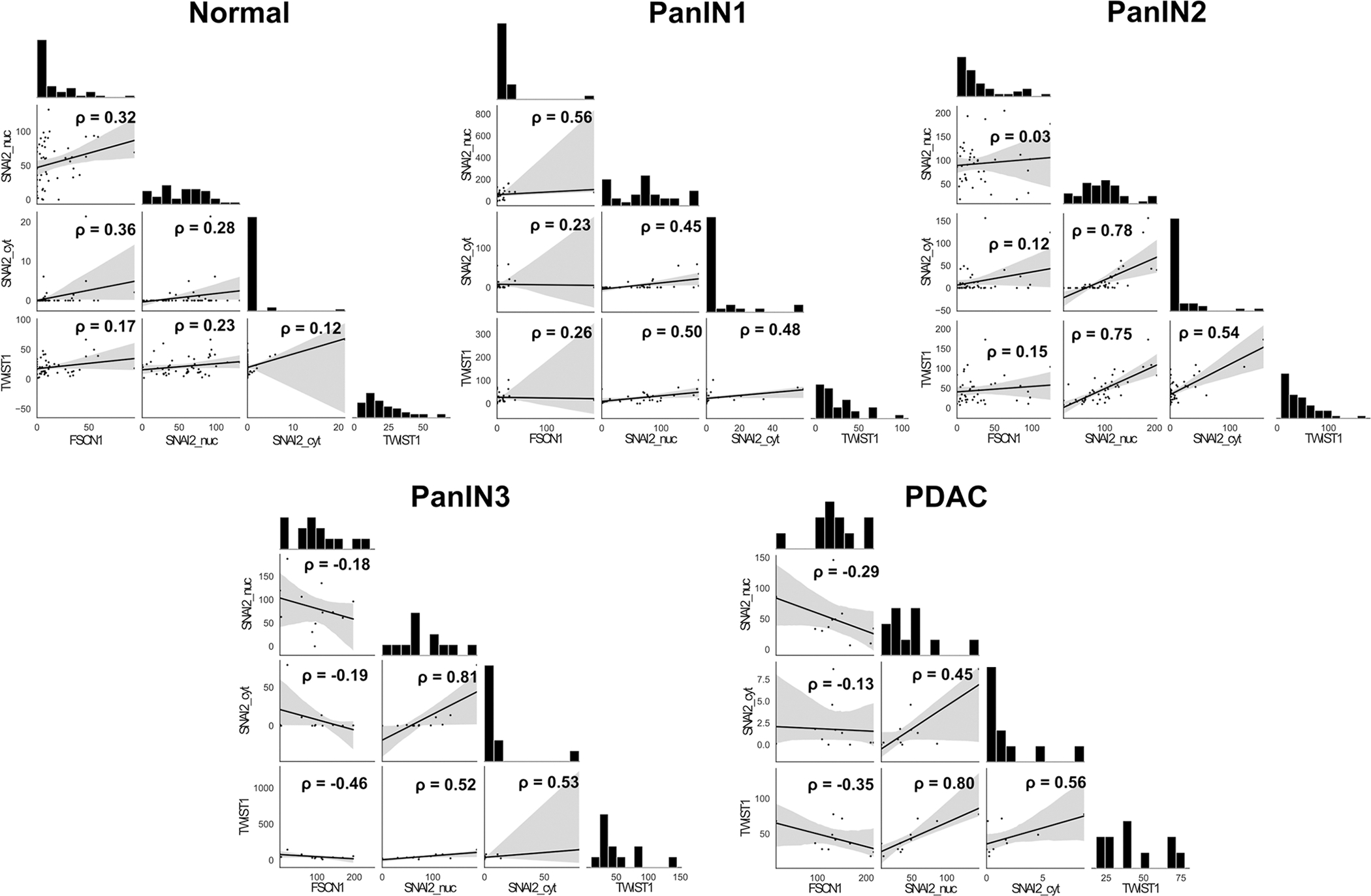
TMA histoscores correlation by duct type. Histograms of expression and Spearman correlation scatterplots with linear regression lines (grey areas represent the 95% confidence intervals) for correlation between histoscores of FSCN1, TWIST1 and SLUG/SNAI2 with Spearman correlation (rho). PanIN1 and PanIN2 are equivalent to low grade PanIN and PanIN3 equivalent to high grade PanIN in the Baltimore consensus classification. Nuc = nuclear, cyt = cytoplasmic.

Analysis was then performed on each category separately (Fig. 4). There was a weak positive correlation in normal ducts between FSCN1 and both nuclear (rho = 0.32, p < 0.05) and cytoplasmic (rho = 0.36, p < 0.05) SLUG/SNAI2. There was moderate correlation between expression of FSCN1 and nuclear SLUG/SNAI2 in PanIN1 ducts (rho = 0.56, p < 0.01). There was no significant correlation in higher grade lesions.

Regrouping the neoplastic ducts as before into low-grade and high-grade lesions (Fig. S4), there was a weak correlation between FSCN1 and nuclear SLUG/SNAI2 expression in low-grade lesions (rho = 0.28, p < 0.05).

### 3.6. FSCN1 and TWIST1 show moderate negative correlation in high-grade ductal lesions

The FSCN1 and TWIST1 h-scores were compared on a core-by-core basis (Figs. 3B and 3 C). Considering all epithelial types together, there was weak correlation between FSCN1 and TWIST1 h-scores (rho = 0.29, p < 0.001).

Analysis was then performed on each category separately (Fig. 4). There was a moderate negative correlation which did not reach statistical significance between FSCN1 and TWIST1 in PanIN3 (rho = −0.46, p = 0.13) and PDAC (rho = −0.35, p = 0.29).

Regrouping neoplastic ducts as before (Fig. S4), there was a moderate negative correlation between the expression of FSCN1 and TWIST1 in high-grade lesions which did not reach statistical significance (rho = −0.37, p = 0.082).

### 3.7. SLUG/SNAI2 and TWIST1 expression correlates in most ductal grades

The SLUG/SNAI2 and TWIST1 h-scores were compared on a core-by-core basis (Fig. 3B and C). Considering all epithelial types together, there was a statistically significant moderate positive correlation between TWIST1 and both nuclear (rho = 0.52, p < 0.001) and cytoplasmic (rho = 0.53, p < 0.001) SLUG/SNAI2 h-scores.

Analysis was then performed on each category separately (Fig. 4). There was a moderate, statistically significant positive correlation between the expression of TWIST1 and both nuclear (rho = 0.5, p < 0.01) and cytoplasmic (rho = 0.48, p < 0.01) SLUG/SNAI2 in PanIN1 ducts. In PanIN2 ducts there was a strong correlation between TWIST1 and nuclear SLUG/SNAI2 (rho = 0.75, p < 0.001) and moderate correlation between TWIST1 and cytoplasmic SLUG/SNAI2 (rho = 0.54, p < 0.001). In PDAC there was a strong correlation between TWIST1 and nuclear SLUG/SNAI2h-scores (rho = 0.8, p < 0.01).

Regrouping neoplastic ducts as before (Fig. S4), there was a moderate and statistically significant positive correlation between the expression of TWIST1 and both nuclear (rho = 0.66, p < 0.001) and cytoplasmic (rho = 0.56, p < 0.001) SLUG/SNAI2 in low-grade lesions. Similarly, in high-grade lesions there was correlation between TWIST1 and both nuclear (rho = 0.61, p < 0.01) and cytoplasmic (rho = 0.54, p < 0.001) SLUG/SNAI2 h-scores.

## 4. Discussion

Fascin is an actin bundling protein that is generally not expressed in normal epithelial cells but associated with cancer progression to malignancy [24]. It enhances actin dynamics and promotes the formation of long thin actin-based protrusions, filopodia, that are used by cells to migrate, invade, and sense their environment (reviewed in [24]). Typically located in the cytoplasm, there have been reports of a nuclear association with unclear physiological significance [48]. In the present study, we only observed cytoplasmic staining of FSCN1 (Fig. 2), although we previously observed occasional nuclear Fscn1 in our mouse model study [12].

We previously demonstrated Fscn1 expression in preneoplastic lesions in mouse pancreata, which increased with grade, and that its expression was associated with that of the EMT-TF SLUG/SNAI2 and was a driver of metastasis [12].

SLUG/SNAI2 represses E-cadherin expression by binding directly to the E-boxes of its promotor sequence [49]. TWIST1 indirectly suppresses E-cadherin by upregulating other EMT-TFs including SLUG/SNAI2 by binding to their respective promotors [50,51]. TWIST1 also upregulates the expression of proteins characteristic of mesenchymal phenotype such as N-cadherin and fibronectin [52].

Here, we investigated the association of FSCN1 with pre-invasive (PanIN) and invasive (PDAC) lesions in a small cohort of human samples from resected PDAC tumours with associated PanIN lesions. While our study has many limitations (described below), it generally supports our findings in mice that FSCN1 is expressed in PanIN and is accompanied by expression of EMT-TFs, such as SLUG//SNAI2, which may be drivers of FSCN1 expression in human PDAC.

Limitations include small sample size and sampling from pancreatectomies performed for established malignancy (PDAC). The ducts classified as normal and PanIN here may not be representative at a subcellular level of morphologically similar ducts in an organ without established malignancy. Cores with assessable ducts contained varying numbers of each grade (Supplementary Table S1), with greater numbers classified as normal or PanIN1–2 compared to PanIN3 and PDAC.

Due to its anatomical location, it is difficult to obtain biopsies from the pancreas [53,54]. The rapid decomposition of the pancreas following death makes obtaining suitable tissue post-mortem technically challenging. As a result, most tissue available for research is obtained from pancreatectomies performed for PDAC and our knowledge of the genetic mechanisms involved in the *development* of PDAC is limited and generally derived from *in vitro* and murine studies. Given the above limitations, it was considered that the material in this TMA was an acceptable compromise to evaluate protein expression in various grades of ductal epithelium in human tissue.

A limitation of any tissue-based study is tumour heterogeneity, which may result in varying expression of proteins throughout a lesion. One way to address this is to include multiple cores from different parts of each tumour in a TMA. TMAs however are vulnerable to tissue loss. In this study, following processing, 77 assessable tissue cores remained, containing tissue sampled from 59 patients. Heterogeneity therefore remains a limitation to data correlating protein expression with patient demographics and clinical outcomes. IHC for each of the proteins was performed on serial sections, so heterogeneity should have minimal influence on the data correlating their relative expression.

An initial *in silico* analysis of a large publicly available dataset of human PDAC was used to identify which EMT-TFs were most relevant to our aim of exploring the relationship between FSCN1 and EMT-TFs in PDAC. We found that high expression of *FSCN1* was associated with poorer overall survival in PDAC, confirming previous findings [12]. *FSCN1* expression correlated with that of EMT-TFs *SLUG/SNAI2*, *TWIST1* and *TCF3*. Due to technical difficulties with the TCF3 antibody and the limited tissue available, we were not able to include TCF3 in the IHC studies.

Using IHC, we confirmed previous reports that there is no/minimal FSCN1 expression in normal ducts [12], while TWIST1 and SLUG/SNAI2 are expressed at low levels [47]. SLUG/SNAI2 and TWIST1 expression peaked in PanIN2. Expression of SLUG/SNAI2 fell slightly in PanIN3 and to a similar level as normal ducts in PDAC, while TWIST1 expression did not significantly change in PanIN3 or PDAC. FSCN1 expression occurred later, achieving significance in PanIN3, and further increased in PDAC. We observed both nuclear and cytoplasmic staining of SLUG/SNAI2 (Fig. 2, S3), likely reflecting the high expression levels and equilibrium between these two cell compartments, but the known function of SLUG/SNAI2 is in the nucleus as a transcription factor.

The results presented here support previous work from our group using *in vitro* and murine models which found that SNAI2 expression was seen in low grade PanIN and induced FSCN1 expression in cells cultured from tumours. We speculated SNAI2 might trigger expression of FSCN1 in higher grade and invasive lesions [12]. In studies from other groups using the KPC mouse model of PDAC, SNAI1 and TWIST1 were found to be redundant in development and metastatic progression of PDAC but were implicated in chemoresistance [55] and ZEB1 was found to be critical for development of PanIN and PDAC [56].

Collectively, these studies provide evidence that results obtained from pre-clinical models are relevant to human disease. Murine models have the advantage that sampling can be undertaken at different time points to examine tumour development at different stages. Future studies may make use of human-derived cell lines and organoids [57] which could be genetically manipulated to replicate EMT *in vitro*.

Our results suggest an evolving role for EMT-TFs during the progression from benign ductal epithelium through non-invasive neoplasia (abnormal cells in which the process of EMT may have started but which have not yet lost E-cadherin expression and thus retain the epithelial property of cohesion) to malignancy (in which the process of EMT is almost or fully complete). However, it is possible that these correlations may not be of biological significance, in particular given the different patterns of expression seen, with sustained increase of FSCN1 in high grade through invasive lesions, while SLUG/SNAI2 and TWIST1 peak in PanIN2, with SLUG/SNAI2 levels then falling in lower grade lesion. Alternatively, there may be a more complex relationship between EMT-TFs and other signalling pathways not explored here. Multiple different signalling pathways could drive EMT in PDAC, resulting in a degree of redundancy such that if one pathway is blocked, for example due to loss or mutation of a gene, EMT can progress via another pathway. There is increasing evidence in the literature of EMT being such a complex, non-linear process [58]. The absence of correlation between survival and expression of these EMT-TFs in PDAC may indicate that once malignancy is established, these proteins have no significant role and other mechanisms regulate cellular level changes which result in poor prognosis.

The results presented here suggest a role for EMT-TFs in the expression of FSCN1 in high-grade PanIN and PDAC and show that higher expression of *FSCN1* is associated with poorer overall survival in human PDAC. Further research is warranted to establish the utility of FSCN1 as a predictive marker in clinical practice and the potential of FSCN1, TWIST1 and SLUG/SNAI2 as early diagnostic markers.

## Supplementary Material

PDAC_FSCN_OS

PDAC_KLF8_OS

Gene_Exp_PDAC

PDAC_SNAI1_OS

PDAC_SNAI2_OS

PDAC_TCF3_OS

PDAC_TWIST1_OS

PDAC_TWIST2_OS

PDAC_ZEB1_OS

PDAC_ZEB2_OS

Supplementary Figure 1

Supplementary Figure 2

Supplementary Figure 3

Supplementary methods, legends tables

Supplementary Figure 4

Appendix A. Supporting information

Supplementary data associated with this article can be found in the online version at doi:10.1016/j.prp.2023.154836.

## Figures and Tables

**Table 1:** Patient Demographics.

	Mean (Range, SD)	

Age at Diagnosis (Years)	64.2 (42 – 82, 6.9)	
Body Mass Index (BMI)	28.8 (20.0 – 43.0, 5.7)	
	**Total (N = 59)**	**(%)**
Sex		
Female	28	(47)
Male	31	(53)
Obesity (BMI >= 30)		
No	35	(66)
Yes	18	(34)
Unknown	6	
Patient Reported Diabetes		
No	46	(78)
Yes	13	(22)
Patient Reported Pancreatitis		
No	31	(65)
Yes	17	
Unknown	11	(35)
Vital Status		
Alive	19	(32)
Deceased	40	(68)

## Data Availability

The datasets supporting the conclusions of this article are included in this published article and its Supplementary material. The results of the *in silico* analysis are in whole based upon data generated by the TCGA Research Network: https://www.cancer.gov/tcga and can be accessed at https://gdc.cancer.gov/about-data/publications/pancanatlas.
