# Supplementary figures and images for: FSCN1 and epithelial mesenchymal transformation transcription factor expression in human pancreatic intraepithelial neoplasia and ductal adenocarcinoma
Source: Pathol Res Pract. Author manuscript; Available in PMC 2025 Jun 17. (PMC12173262; doi:10.1016/j.prp.2023.154836)

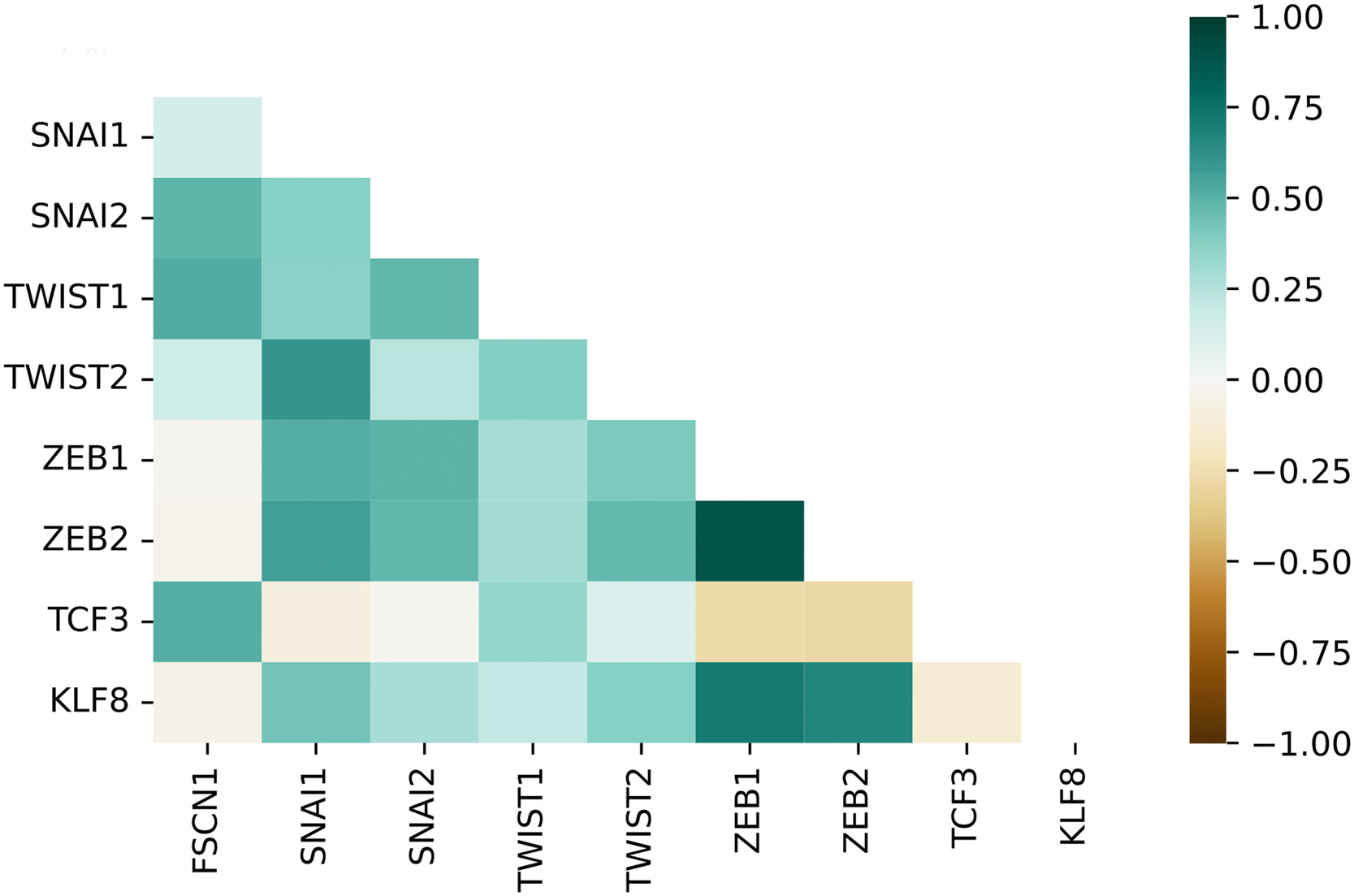

Supplement: Supplementary Figure 1 [file NIHMS2084962-supplement-Supplementary_Figure_1.jpg]

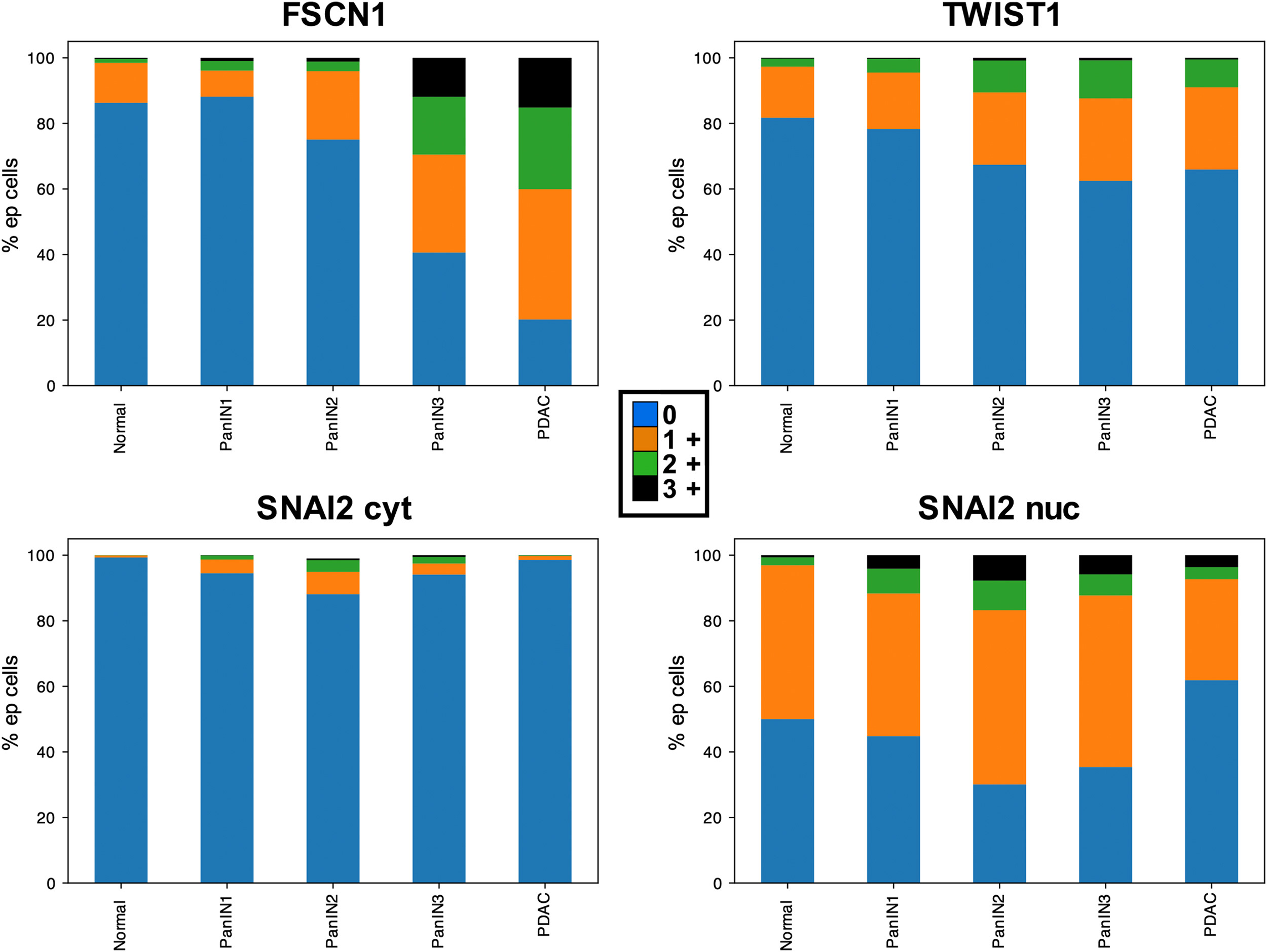

Supplement: Supplementary Figure 2 [file NIHMS2084962-supplement-Supplementary_Figure_2.jpg]

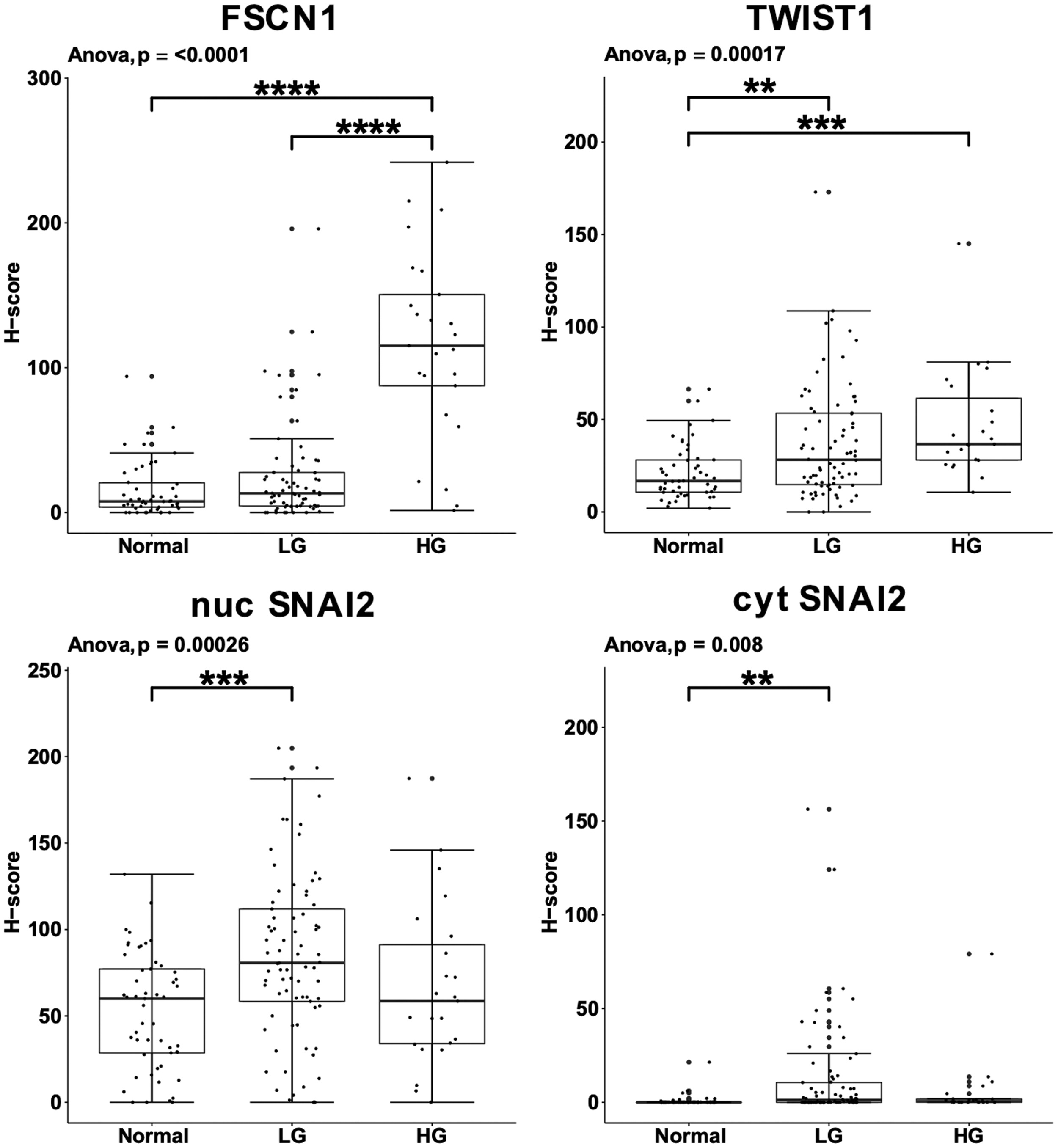

Supplement: Supplementary Figure 3 [file NIHMS2084962-supplement-Supplementary_Figure_3.jpg]

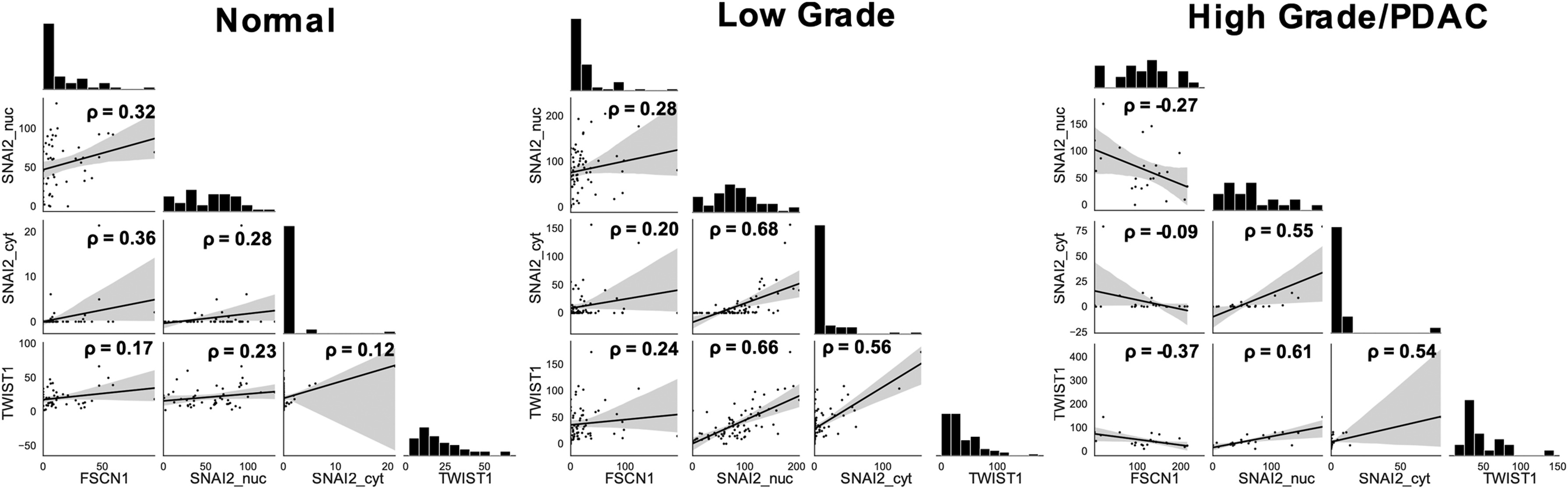

Supplement: Supplementary Figure 4 [file NIHMS2084962-supplement-Supplementary_Figure_4.jpg]
