# Supplementary material for: FSCN1 and epithelial mesenchymal transformation transcription factor expression in human pancreatic intraepithelial neoplasia and ductal adenocarcinoma
Source: Pathol Res Pract. Author manuscript; Available in PMC 2025 Jun 17. (PMC12173262; doi:10.1016/j.prp.2023.154836)
Supplement: Supplementary methods, legends tables [file NIHMS2084962-supplement-Supplementary_methods__legends_tables.docx]

**Supplementary Materials and Methods**

**Immunohistochemistry**

Sections were washed in xylene and rehydrated by immersion in sequential graded ethanol solutions. Antigen retrieval was achieved by immersion in PT module antigen unmasking solution (FSCN1: citrate based solution, Vector Labs H-3300. SNAI2, TCF3 and TWIST1: high pH solution, Agilent K8004) at 97^o^C for 20 minutes, followed by washing in Flex wash buffer (Agilent K8007), then exposure to block endogenous peroxidase (Agilent S2023) for 5 minutes. For all except FSCN1, there was an additional blocking step of 20 minutes exposure to M.O.M (Mouse on Mouse) blocking reagent. Following a further wash in buffer, the slides were then incubated in primary antibody (diluted in Primary Antibody diluent, Agilent, S2022) for 35 minutes, then the appropriate secondary antibody (Mouse EnVision, Agilent K4001; Rabbit EnVision, Agilent K4003) for 30 minutes. Following that, they were exposed to 3,3’Diaminobenzidine tetrahydrochloride (Agilent K3468) for 10 minutes, washed in deionised water, then stained with haematoxylin Z (CellPath RBA-4201-00A), washed, then dehydrated and mounted.

**Histoscore Calculation**

Whole slide images were managed using the TMA add-on module in Halo image analysis platform v3.1 (Indica Labs). Utilising the layer annotation tool, ducts were assigned to separate layers according to classification (normal, PanIN1, PanIN2, PanIN3 or PDAC).

The Cytonuclear IHC module was used to quantify expression of FSCN1, SNAI2 and TWIST1 within each layer. The cut-off staining optic density values were assigned by the pathologist and are detailed in Supplementary Table 1. Each cell was assigned a score between 0 and 3 based on the optical density of the IHC counterstain (DAB), for both the nuclear and cytoplasmic compartments. The module calculated a histoscore (h-score) for each layer, a product of the percentage of positive cells and their intensity scores [1].

Some spots on the TMA contained no tissue, either because the core was exhausted, or the material had been lost in processing. Some of the tissue cores contained no ducts or the material was folded such that the ducts could not be graded. These cores were excluded from the analysis. In some cores ducts of a certain grade were not represented in all sections and in such cases comparison between the h-scores for different immunostains could not be performed.

**Reference:**

[1] H. Goulding, S. Pinder, P. Cannon, *et al.* A new immunohistochemical antibody for the assessment of estrogen receptor status on routine formalin-fixed tissue samples. *Hum Pathol*. 1995; 26(3): 291-4. Epub 1995/03/01. doi: 10.1016/0046-8177(95)90060-8. PubMed PMID: 7890280.

**Supplementary Figure Legends**

**Figure S1 - correlation of *FSCN1* and EMT transcription factors in human PDAC.**

Heatmap showing correlation of expression of *FSCN1* and EMT transcription factors data based on data extracted from cBioPortal (related to Figure 1A).

**Figure S2 – Immunohistochemistry of FSCN1, SLUG/SNAI2 and TWIST1 in human pancreas, PanIN and PDAC.**

Stacked bar charts showing percentage of ductal epithelial cells showing negative (0), weak (1+), moderate (2+) and strong (3+) IHC staining for FSCN1, TWIST1 and SLUG/SNAI2. PanIN1 and PanIN2 are equivalent to low grade PanIN and PanIN3 equivalent to high grade PanIN in the Baltimore consensus classification. Cyt = cytoplasmic, nuc = nuclear.

**Figure S3 – TMA histoscores ANOVA by revised groups.**

Tukey boxplots (minimum value, first quartile, median, third quartile, maximum value), of IHC histoscores by duct type with results of ANOVA analysis (n = 11-52, see Supplementary Table S1 for numbers of cores assessed per grade). LG = low grade (equivalent to PanIN 1-2 in the 2010 WHO classification), HG = high grade (PanIN3 in the WHO classification)/invasive (PDAC), nuc = nuclear, cyt = cytoplasmic. **p<0.01, ***p<0.001, ****p<0.0001 (Tukey’s post-hoc test).

**Figure S4 - TMA histoscores correlation by revised groups.**

Histograms of expression and Spearman correlation scatterplots with linear regression lines (grey areas represent the 95% confidence intervals) for correlation between histoscores of FSCN1, TWIST1 and SLUG/SNAI2 with Spearman correlation (rho). LG = low grade (equivalent to PanIN 1-2 in the 2010 WHO classification), HG = high grade (PanIN3 in the WHO classification)/invasive (PDAC), nuc = nuclear, cyt = cytoplasmic.

**Supplementary Tables**

## Supplementary Table S1. Number of TMA Cores per Duct Grade

## Supplementary Table S2. Halo settings

**Supplementary Table S3. Patient Data by Protein Histoscores**

**Supplementary Table S1. Number of TMA Cores per Duct Grade**

| **Stain** | **FSCN1** | **SNAI2 - nuc** | **SNAI2 - cyt** | **TWIST1** |
| --- | --- | --- | --- | --- |
| Normal | 50 | 52 | 52 | 50 |
| PanIN1 | 25 | 32 | 32 | 31 |
| PanIN2 | 41 | 45 | 45 | 47 |
| PanIN3 | 14 | 12 | 12 | 12 |
| PDAC | 11 | 11 | 11 | 11 |

## Supplementary Table S2. Halo settings

|  | **FSCN1** | **SNAI2 cyt** | **SNAI2 nuc** | **TWIST1** |
| --- | --- | --- | --- | --- |
| Maximum Cytoplasm Radius | 2.58 | 2.58 | 2.58 | 2.58 |
| Maximum Nuclear Size | 115.995 | 115.995 | 115.995 | 115.995 |
| Minimum Nuclear OD | 0.146 | 0.146 | 0.146 | 0.146 |
| Minimum Nuclear Roundness | 0.245 | 0.245 | 0.245 | 0.245 |
| Minimum Nuclear Size | 22.1247 | 22.124 | 22.124 | 8.850 |
| Minimum Tissue OD | 0.06 | 0.06 | 0.06 | 0.06 |
| Nuclear Contrast Threshold | 0.457 | 0.457 | 0.457 | 0.457 |
| Nuclear Segmentation Aggressiveness | 1 | 1 | 1 | 1 |
| Nuclear Stain | 0.482,0.587,0.293 | 0.482,0.587,0.293 | 0.482,0.587,0.293 | 0.555,0.600,0.353 |
| Positive Stain | 0.467,0.614,0.674 | 0.467,0.614,0.674 | 0.219,0.361,0.498 | 0.708,1.009,1.128 |
| Stain Localization | Cytoplasm | Nucleus | Cytoplasm | Nucleus |
| Stain Min OD 1+ | 0.097 | 0.150 | 0.139 | 0.172 |
| Stain Min OD 2+ | 0.241 | 0.400 | 0.226 | 0.227 |
| Stain Min OD 3+ | 0.392 | 0.500 | 0.410 | 0.332 |

## Supplementary Table S3. Patient Data by Protein Histoscores

| **FSCN1** | Missing (N=2) | h-score below median (N=28) | h-score above median (N=29) | Total (N=59) | p value |
| --- | --- | --- | --- | --- | --- |
| **Survival (Surgery) Events** | 2 | 19 | 19 | 40 | 0.3710 |
| Median Survival Days (95% CI) | 938.0 (398.0- ) | 1380.0 (870.0-2315.0) | 659.0 (495.0-1037.0) | 951.0 (659.0-1380.0) |  |
| **Age at Diagnosis** |  |  |  |  |  |
| Mean (SD) | 61.5 (10.6) | 64.2 (10.3) | 64.4 (9.2) | 64.3 (9.7) | 0.8982 |
| Median (95% CI) | 61.5 (54.0- ) | 64.0 (60.0-69.0) | 62.0 (60.0-66.0) | 62.0 (60.0-66.0) | 0.8347 |
| Q1, Q3 | 54.0, 69.0 | 59.0, 71.0 | 58.0, 73.0 | 58.0, 72.0 |  |
| Range | (54.0-69.0) | (42.0-82.0) | (48.0-82.0) | (42.0-82.0) |  |
| **Sex** |  |  |  |  | 0.2359 |
| Female | 2 | 15 (53.6%) | 11 (37.9%) | 26 (45.6%) |  |
| Male | 0 | 13 (46.4%) | 18 (62.1%) | 31 (54.4%) |  |
| **Obesity (BMI >= 30)** |  |  |  |  | 0.6920 |
| Missing | 0 | 2 | 4 | 6 |  |
| No | 1 | 18 (69.2%) | 16 (64.0%) | 34 (66.7%) |  |
| Yes | 1 | 8 (30.8%) | 9 (36.0%) | 17 (33.3%) |  |
| **Patient Reported Diabetes** |  |  |  |  | 0.3815 |
| No | 2 | 23 (82.1%) | 21 (72.4%) | 44 (77.2%) |  |
| Yes | 0 | 5 (17.9%) | 8 (27.6%) | 13 (22.8%) |  |
| **Patient Reported Pancreatitis** |  |  |  |  | 0.3454 |
| Missing | 0 | 5 | 6 | 11 |  |
| No | 0 | 17 (73.9%) | 14 (60.9%) | 31 (67.4%) |  |
| Yes | 2 | 6 (26.1%) | 9 (39.1%) | 15 (32.6%) |  |
| **SLUG/SNAI2 nuclear** | Missing (N=1) | h-score below median (N=29) | h-score above median (N=29) | Total (N=59) | p value |
| **Survival (Surgery) Events** | 1 | 16 | 23 | 40 | 0.8260 |
| Median Survival Days (95% CI) | 398.0 ( - ) | 831.0 (528.0-1317.0) | 1192.0 (659.0-1681.0) | 951.0 (659.0-1380.0) |  |
| **Age at Diagnosis** |  |  |  |  |  |
| Mean (SD) | 69.0 ( ) | 63.6 (8.7) | 64.7 (10.7) | 64.1 (9.7) | 0.5642 |
| Median (95% CI) | 69.0 ( - ) | 61.0 (60.0-66.0) | 63.0 (58.0-70.0) | 62.0 (60.0-66.0) | 0.6148 |
| Q1, Q3 | 69.0, 69.0 | 60.0, 66.0 | 57.0, 73.0 | 58.0, 72.0 |  |
| Range | (69.0-69.0) | (48.0-82.0) | (42.0-82.0) | (42.0-82.0) |  |
| **Sex** |  |  |  |  | 0.7924 |
| Female | 1 | 14 (48.3%) | 13 (44.8%) | 27 (46.6%) |  |
| Male | 0 | 15 (51.7%) | 16 (55.2%) | 31 (53.4%) |  |
| **Obesity (BMI >= 30)** |  |  |  |  | 1.0000 |
| Missing | 0 | 3 | 3 | 6 |  |
| No | 1 | 17 (65.4%) | 17 (65.4%) | 34 (65.4%) |  |
| Yes | 0 | 9 (34.6%) | 9 (34.6%) | 18 (34.6%) |  |
| **Patient Reported Diabetes** |  |  |  |  | 0.7529 |
| No | 1 | 23 (79.3%) | 22 (75.9%) | 45 (77.6%) |  |
| Yes | 0 | 6 (20.7%) | 7 (24.1%) | 13 (22.4%) |  |
| **Patient Reported Pancreatitis** |  |  |  |  | 0.0804 |
| Missing | 0 | 2 | 9 | 11 |  |
| No | 0 | 15 (55.6%) | 16 (80.0%) | 31 (66.0%) |  |
| Yes | 1 | 12 (44.4%) | 4 (20.0%) | 16 (34.0%) |  |
| **SLUG/SNAI2 cytoplasmic** | Missing (N=1) | h-score below median (N=29) | h-score above median (N=29) | Total (N=59) | p value |
| **Survival (Surgery) Events** | 1 | 19 | 20 | 40 | 0.6140 |
| Median Survival Days (95% CI) | 398.0 ( - ) | 951.0 (608.0-1380.0) | 786.0 (495.0-1681.0) | 951.0 (659.0-1380.0) |  |
| **Age at Diagnosis** |  |  |  |  |  |
| Mean (SD) | 69.0 ( ) | 65.2 (10.1) | 63.0 (9.3) | 64.1 (9.7) | 0.4450 |
| Median (95% CI) | 69.0 ( - ) | 62.0 (60.0-70.0) | 62.0 (58.0-66.0) | 62.0 (60.0-66.0) | 0.2487 |
| Q1, Q3 | 69.0, 69.0 | 60.0, 75.0 | 57.0, 68.0 | 58.0, 72.0 |  |
| Range | (69.0-69.0) | (48.0-82.0) | (42.0-80.0) | (42.0-82.0) |  |
| **Sex** |  |  |  |  | 0.7924 |
| Female | 1 | 14 (48.3%) | 13 (44.8%) | 27 (46.6%) |  |
| Male | 0 | 15 (51.7%) | 16 (55.2%) | 31 (53.4%) |  |
| **Obesity (BMI >= 30)** |  |  |  |  | 0.7029 |
| Missing | 0 | 4 | 2 | 6 |  |
| No | 1 | 17 (68.0%) | 17 (63.0%) | 34 (65.4%) |  |
| Yes | 0 | 8 (32.0%) | 10 (37.0%) | 18 (34.6%) |  |
| **Patient Reported Diabetes** |  |  |  |  | 0.7529 |
| No | 1 | 23 (79.3%) | 22 (75.9%) | 45 (77.6%) |  |
| Yes | 0 | 6 (20.7%) | 7 (24.1%) | 13 (22.4%) |  |
| **Patient Reported Pancreatitis** |  |  |  |  | 0.1814 |
| Missing | 0 | 6 | 5 | 11 |  |
| No | 0 | 13 (56.5%) | 18 (75.0%) | 31 (66.0%) |  |
| Yes | 1 | 10 (43.5%) | 6 (25.0%) | 16 (34.0%) |  |
| **TWIST1** | Missing (N=2) | h-score below median (N=28) | h-score above median (N=29) | Total (N=59) | p value |
| **Survival (Surgery) Events** | 2 | 18 | 20 | 40 | 0.7051 |
| Median Survival Days (95% CI) | 331.5 (265.0- ) | 951.0 (608.0-1478.0) | 1037.0 (495.0-1601.0) | 951.0 (659.0-1380.0) |  |
| **Age at Diagnosis** |  |  |  |  |  |
| Mean (SD) | 67.5 (2.1) | 64.5 (8.4) | 63.7 (11.0) | 64.1 (9.8) | 0.7980 |
| Median (95% CI) | 67.5 (66.0- ) | 61.0 (60.0-66.0) | 62.0 (57.0-70.0) | 62.0 (60.0-66.0) | 0.9887 |
| Q1, Q3 | 66.0, 69.0 | 60.0, 69.5 | 57.0, 75.0 | 58.0, 72.0 |  |
| Range | (66.0-69.0) | (48.0-82.0) | (42.0-82.0) | (42.0-82.0) |  |
| **Sex** |  |  |  |  | 0.9034 |
| Female | 2 | 13 (46.4%) | 13 (44.8%) | 26 (45.6%) |  |
| Male | 0 | 15 (53.6%) | 16 (55.2%) | 31 (54.4%) |  |
| **Obesity (BMI >= 30)** |  |  |  |  | 0.7823 |
| Missing | 0 | 4 | 2 | 6 |  |
| No | 2 | 16 (66.7%) | 17 (63.0%) | 33 (64.7%) |  |
| Yes | 0 | 8 (33.3%) | 10 (37.0%) | 18 (35.3%) |  |
| **Patient Reported Diabetes** |  |  |  |  | 0.3815 |
| No | 2 | 23 (82.1%) | 21 (72.4%) | 44 (77.2%) |  |
| Yes | 0 | 5 (17.9%) | 8 (27.6%) | 13 (22.8%) |  |
| **Patient Reported Pancreatitis** |  |  |  |  | 0.6861 |
| Missing | 0 | 4 | 7 | 11 |  |
| No | 1 | 15 (62.5%) | 15 (68.2%) | 30 (65.2%) |  |
| Yes | 1 | 9 (37.5%) | 7 (31.8%) | 16 (34.8%) |  |
